# Inkjet Printing of Polyacrylic Acid-Coated Silver Nanoparticle Ink onto Paper with Sub-100 Micron Pixel Size

**DOI:** 10.3390/ma12142277

**Published:** 2019-07-15

**Authors:** Arunakumari Mavuri, Andrew G. Mayes, Matthew S. Alexander

**Affiliations:** 1 Engineering, Faculty of Science, University of East Anglia, Norwich NR4 7TJ, UK; 2 School of Chemistry, University of East Anglia, Norwich NR4 7TJ, UK

**Keywords:** inkjet printing, silver nanoparticles, conductive inks, printed electronics, low-temperature sintering

## Abstract

Printed electronics (PE) technology shows huge promise for the realisation of low-cost and flexible electronics, with the ability to pattern heat- or pressure-sensitive materials. In future developments of the PE market, the ability to produce highly conductive, high-resolution patterns using low-cost and roll-to-roll processes, such as inkjet printing, is a critical technology component for the fabrication of printed electronics and displays. Here, we demonstrate inkjet printing of polyacrylic acid (PAA) capped silver nanoparticle dispersions onto paper for high-conductivity electronic interconnects. We characterise the resulting print quality, feature geometry and electrical performance of inkjet patterned features and demonstrate the high-resolution printing, sub-100 micron feature size, of silver nanoparticle materials onto flexible paper substrate. Printed onto photo-paper, these materials then undergo chemically triggered sintering on exposure to chloride contained in the paper. We investigated the effect of substrate temperature on the properties of printed silver material from room temperature to 50 °C. At room temperature, the resistivity of single layer printed features, of average thickness of 500 nm and width 85 µm, was found to be 2.17 × 10^−7^ Ω·m or 13 times resistivity of bulk silver (RBS). The resistivity initially decreased with an increase in material thickness, when achieved by overprinting successive layers or by decreasing print pitch, and a resistivity of around 10 times RBS was observed after overprinting two times at pitch 75 µm and with single pass print pitch of between 60 and 80 µm, resulting in line thickness up to 920 nm. On further increases in thickness the resistivity increased and reached 27 times RBS at print pitch of 15 µm. On moderate heating of the substrate to 50 °C, more compact silver nanoparticle films were formed, reducing thickness to 200 nm from a single pass print, and lower material resistivity approaching five times RBS was achieved.

## 1. Introduction

Printed electronics (PE) technology has captured considerable attention in recent years for its wide range of advantages over traditional subtractive technologies, such as ease of production, cost-effectiveness, applicability to large-scale production and its versatility towards substrates, with the ability to pattern heat- or pressure-sensitive substrates. Furthermore, PE techniques are purely additive in nature and reduce material waste and consume fewer chemicals in the process, bringing environmental benefits. In recent years, huge research effort has been invested in developing PE technology for device fabrication in various applications. Currently, the main focus of study is on devices like flexible batteries [1,2], electro-optic devices [3,4], organic photovoltaics [5,6], logic and memory components such as thin film transistors (TFTs) [7,8], sensors [9] and radio frequency identification (RFID) tags [10,11]. PE utilises various printing equipment to develop electronic devices on different substrates like glass, polyethylene terephthalate (PET), paper and polyimide (PI). Of these, paper has received much attention as a flexible substrate due to its low cost, environmental benefits of recyclability, and its biodegradable nature [12].

Most target applications for PE incorporate conducting layers and/or conducting patterned features. These conductive materials can be based on conductive polymers [13], carbon-based materials [14], or metallic nanoparticles [15,16]. To date, metallic nanoparticle (NP) inks have proven the most suitable due to their high-conductivity and have received the most attention. Metallic NP inks are typically composed of Cu, Ag or Au. Au has high conductivity and thermal stability but is too expensive for mass production [17] and whilst copper is the most economical, the ease of oxidation in air decreases the conductivity. Hence, silver is the most studied material for NP inks to date due to its good oxidation resistance to air, high conductivity and lower cost.

For large-scale manufacturing, low-cost flexible substrates, such as paper or plastic, are desired but these materials are thermally sensitive and require low-temperature sintering, typically limiting process temperatures to below 150 °C. In future developments of the PE market, the ability to produce highly conductive, high-resolution patterns using low-cost and roll-to-roll processes, such as inkjet printing, on flexible, thermally sensitive substrates is one of the most critical technology components for the fabrication of printed electronics and displays. To this end, the development of metallic NP inks can bring about a decrease in the sintering temperature required. However, due to the reactive nature of the metal nanoparticles it is necessary to coat or cap the metal with polymer stabilisers to enhance dispersion in the solvent system and prevent oxidation prior to sintering. A wide range of metal nanoparticles have been investigated for PE applications and the preferable size of the nanoparticles in inkjet ink formulation is 5–100 nm. In order to fuse the nanoparticle after printing to produce a highly conductive material it is necessary to remove the organic capping agents. This is typically achieved by thermal or chemical sintering methods. The typical temperature required for NP ink to decompose the organic stabilizers is above 200 °C, rendering the use of paper and most plastic substrate materials impossible. Chemical sintering approaches have recently been developed for polyacrylic acid (PAA) and polyvinyl pyrrolidone (PVP) capped AgNPs in which simple exposure to chloride ions could be used to replace and detach the organic stabiliser from the AgNPs [18,19]. On de-bonding of the stabiliser, the Ag NPs are allowed to come in close contact and to undergo coalescence to produce highly conductive silver deposits.

Today, huge research effort is being invested in the development of conductive inks for inkjet printing, where proper selection of the ink’s physical properties is the key factor for achieving high quality printing. Improper selection of ink systems leads to unstable ink-jetting and satellite production or splashing on impact and a poor-quality print. The major parameters that affect the drop performance and size are viscosity, density and surface tension. A key challenge now lies in the development of stable inkjet formulations to enable fine pattern deposition of the AgNP material and application in printed electronics.

In early work [18] on room temperature sintered AgNP inks, excellent conductivity of inkjet-printed patterns of a water based silver nanoparticle dispersion was achieved by chemical sintering on contact with poly(diallyldimethylammonium chloride) however, the feature size demonstrated was not well controlled and comparatively large and of the order 500 µm, likely due to satellite drop formation. In later work [20,21], PAA capped AgNPs were dispersed in water/ethylene glycol mixtures and inkjet-printed onto photo paper. Room temperature printed material showed a resistivity between 12–100 µΩ·cm and good conductivity was achieved when the patterns were overprinted many times and then thermally sintered at 120 °C or above, resulting in a minimum of around 2–3 times resistivity of bulk silver (RBS). However, in both studies the printed features showed poor quality due to satellite drop production and overprinting was necessary to achieve a continuous track of good conductivity. Another group [22] investigated the effect on electrical properties, using a commercial AgNP inkjet formulation, of different active coatings on paper substrates to initiate chemical sintering. The printed features were well defined and of 400 µm width and most exhibited moderate conductivity at room temperature that was enhanced as thermal sintering was performed up to 180 °C, however it was found that one coating showed excellent conductivity at a low temperature of 60 °C. Recent work [23] also investigated the inkjet printing of Ag NP inks for low-temperature conductive patterns on paper substrates, and after overprinting more than 10 times and heating the subsequent features to 100 °C or above, a good conductivity was achieved, but the feature size produced was very large and in excess of 1mm.

In the present work, we report the development of PAA-coated silver nanoparticle dispersions for inkjet printing based on simple solvent mixtures containing no surfactants or other additives. These formulations enabled stable inkjet onto flexible paper substrate. We characterise the resulting print quality and electrical performance of inkjet patterned features and demonstrate the capability of highly controlled printing, with sub-100 micron feature size, of silver nanoparticle materials onto a photo-paper substrate. These patterns then undergo chemically triggered, low-temperature sintering to produce interconnects with a resulting resistivity approaching five times bulk silver.

## 2. Results and Discussion

### 2.1. Silver Nanoparticle Formulation Properties

**Figure 1:**
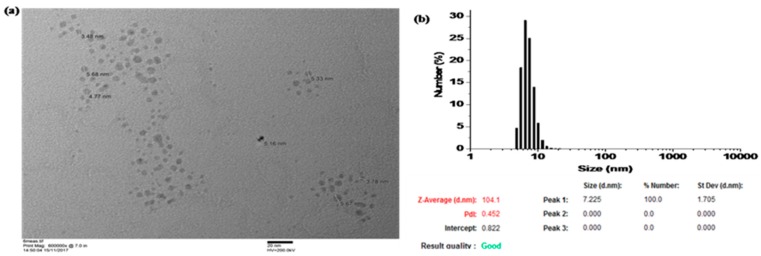
(**a**) TEM image of the solid AgNPs and (**b**) nanoparticle size distribution.

The as prepared AgNPs were dispersed in water to measure the UV-Vis absorption peak, which was found to be at 408 nm, indicating the formation of silver nanoparticles with a size range of approximately 1–30 nm [24]. The size was further studied from TEM images, see Figure 1a, and nanoparticles predominantly where a mean particle diameter of 7.2 nm was observed, as shown in Figure 1b.

Initial testing was performed with water/alcohol mixtures to reduce surface tension, but poor print quality and satellite drop formation was observed due to rapid drying of the ink on the nozzle surface. In order to overcome this issue and reduce drying speed, ethylene glycol was added to produce a tri-solvent system. Once dispersed in the water/propanol/ethylene glycol tri-solvent inkjet mixture, the mean particle diameter from DLS measurements and zeta potential were determined to be 28 nm and −39 mV respectively. The results presented in this paper are from inkjet printing from a 30% (w/w) AgNP suspension in tri-solvent ink where the physical properties of the water/ethylene glycol/propanol ink were determined as a density of 1.28 g/cm^3^, viscosity of 7 cPs and surface tension of 27.1 dynes/cm.

### 2.2. Inkjet Printing Setup

**Figure 2:**
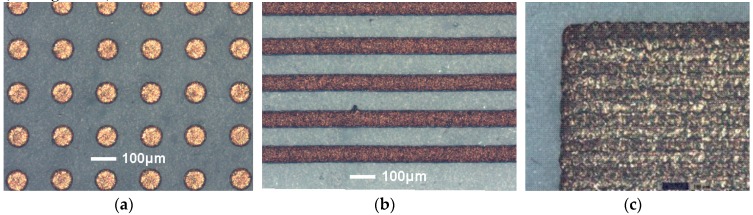
Optical images of the printed (**a**) dot arrays, (**b**) lines at 65 µm print pitch and (**c**) pads on photo paper with a nozzle diameter 50 µm.

The inkjet printing performance of the 30% AgNP in tri-solvent ink was investigated using a Jetlab 4 inkjet printer and a glossy photo-paper substrate with a nozzle aperture size of 50 µm and 80 µm. Printing parameters such as dwell voltage, time and back pressure were adjusted in order to achieve a stable single droplet ejection with minimal volume, as observed by strobe inspection facility. Once stable jetting had been achieved, the print pitch between droplets was adjusted initially to separate the printed features in order to determine dot size and then adjusted to overlap the features to produce lines and 2D patterned features of controlled geometry. As an example, the resulting printed features on glossy photo paper substrate exhibited a diameter of around 90 µm from an 80 µm nozzle and a feature size of around 80 µm from a 50 µm nozzle. An example of a printed dot array, lines and 2D pads from a 50 µm nozzle is shown in Figure 2a–c. From the optical images in Figure 2, one can see a good quality of print with consistent dot shape and size and with the absence of satellite drop formation. By the selection of an appropriate print pitch the droplets can form continuous lines with ideal characteristics and virtually no irregularity or bulging (see Figure 2b).

**Figure 3:**
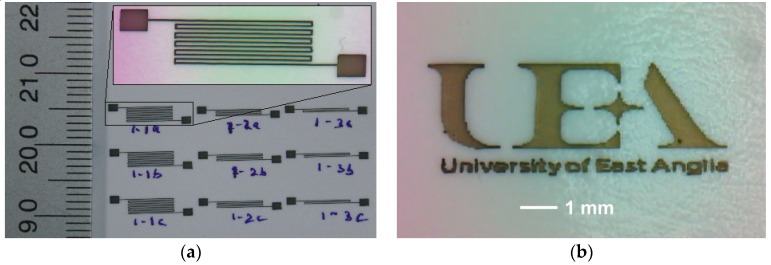
Optical images of (**a**) printed 2D circuit patterns and pads alongside ruler scale for reference and (**b**) University of East Anglia logo printed with feature size 85 µm and dot spacing 65 µm with a nozzle diameter 50 µm.

In order to demonstrate the printability of the ink formulation, a printed 2D pattern with pads and University of East Anglia logo was produced on glossy paper with a pixel size of <100 µm as in Figure 3a,b.

### 2.3. Effect of Print Pitch on Geometry and Electrical Properties with Room Temperature Sintering

**Figure 4:**
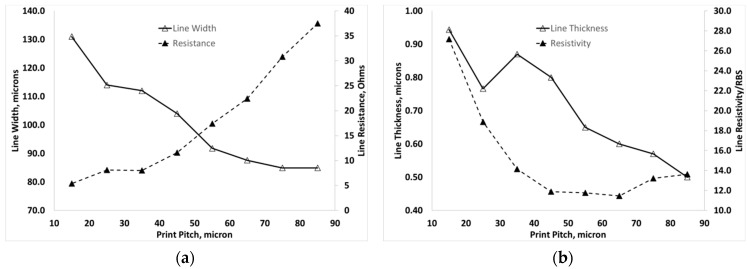
Results from room-temperature printed lines on glossy paper with a 50 µm nozzle diameter (**a**) Effect of print pitch on line width and resistance and (**b**) the effect of print pitch on line thickness and resistivity shown relative to resistivity of bulk silver (RBS).

To investigate the effect of print pitch (dot spacing) on the geometry and electrical properties of the printed features, the print pitch between droplets was varied over the range 85 µm to 15 µm and 10 replica lines were printed in a single pass for each condition. For a 50 µm nozzle with paper substrate at room temperature, the effect of print pitch on the line width and electrical resistance and the effect of print pitch on line thickness and resistivity are shown in Figure 4a,b, respectively.

**Figure 5:**
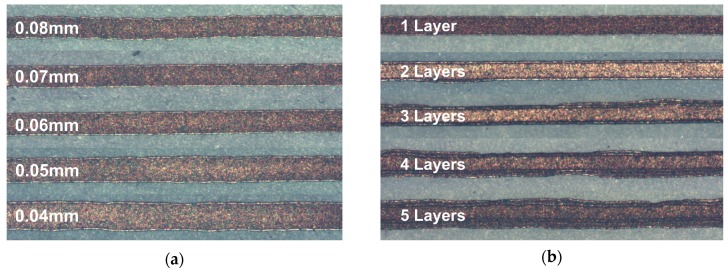
Optical micrograph images showing variation of line width of room-temperature printed lines on photo paper from a 50 µm nozzle by (**a**) increasing pitch of single pass print from 80 µm to 40 µm and (**b**) overprinting of layers up to five times at pitch 65 µm.

Line width was found to increase as the print pitch was decreased from a minimum of 84.9 µm at print pitch 85 µm to more than 130 µm at 15 µm pitch, due to the increased amount of lateral spreading prior to drying as shown in Figure 5a. The resistance of the lines decreased with decreasing pitch from 37.5 Ω at 85 µm to 5.4 Ω at 15 µm pitch. From Figure 4b, the thickness was also found to increase as the pitch decreased and the resistivity was initially found to be largely independent of dot spacing over the range 85 µm to 45 µm but on further decreasing the print pitch the resistivity of printed features increased up to a pitch of 15 µm. The resistivity at the lower pitch range was found to be 1.8–2.0 × 10^−7^ Ω·m, which is around 11–13 times RBS. On decreasing the pitch beyond 45 µm, the resistivity continues to increase, reaching around 30 times RBS at 15 µm. These results indicate that there is an optimum print pitch range around 75–65 µm where the resistivity is near minimum and the dimensions of the printed silver tracks have not significantly increased.

### 2.4. Comparison of Layer Overprinting and Print Pitch Variation on Properties

**Figure 6:**
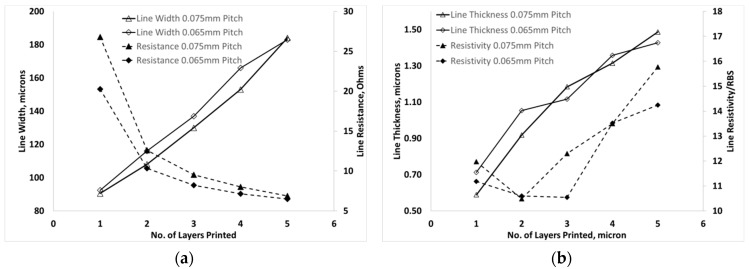
Properties of room-temperature overprinted features on photo paper from a 50 µm nozzle at 65 µm and 75 µm print pitch, showing the dependence of (**a**) line width and resistance on number of printed layers and (**b**) line thickness and resistivity on number of printed layers.

To determine if the geometry and electrical performance of printed lines were influenced by an overprinting approach rather than a single pass printing method, a fixed pitch was selected at 65 µm and 75 µm and continuous lines were overprinted up to five times on photo paper. An example optical micrograph image showing effect of increasing pitch on the geometry of printed track in shown in Figure 5a and in contrast, example images showing effect of successive layer overprinting at 65 µm print pitch is shown in Figure 5b. It can be seen that the overprinting approach results in additional lateral spreading in successively printed features and results in a less uniform feature. The thickness and width of the printed lines both increase with overprinting from a thickness of around 550 nm and width 90 µm with a single layer at 65 µm pitch to a thickness of 1.4 µm and width 184 µm after overprinting to achieve five layers. In a similar observation to results from print pitch variation, the resistivity of the overprinted layers is largely unchanged at between 1.8–2.1 × 10^−7^ Ω·m, around 12 times RBS, up until 3–4 layers are printed, after which a steady increase in resistivity is observed, as shown in Figure 6. The threshold for increasing resistivity appears to correspond with a material thickness of around 1 µm and the resistivity increased to over 2.5 × 10^−7^ Ω·m, approaching 16 times RBS, when five layers were printed. In single-pass printing the resistivity was found to increase when the thickness exceeded around 0.8 µm. This seems likely to be a result of less effective chemical sintering due to depletion of the available chloride for sintering on increasing silver material loading. In comparison to other work [21], the thickness of the inkjet-printed PAA-coated AgNPs onto paper was measured and found to increase non-linearly with the number of overprinted layers up to 14 times. In [21], the ink required a minimum of two layers before continuity in the tracks, where the thickness was found to be approximately 100 nm due to a disperse print area of > 0.5 mm due to unstable printing and presence of satellite droplets. However, on increasing the number of layers to 10 the thickness approached 500 nm, which was comparable to that observed from a single-pass print in the current work. The room-temperature resistivity with 10 printed [21] layers was found to be 1.0 × 10^−7^ Ω·m or 7 times RBS, which is close to the minimum value of 10 times RBS observed in the current work.

### 2.5. Effect of Substrate Temperature on Material Properties

To determine if the substrate temperature during printing would influence the printed material geometry and electrical performance, a series of printed test patterns were produced by both single-pass printing at varying pitch and by successive layer overprinting at a fixed print pitch with an elevated substrate temperature of 40 °C and 50 °C, achieved using the integrated platen heater provided on the Jetlab 4 inkjet system.

**Figure 7:**
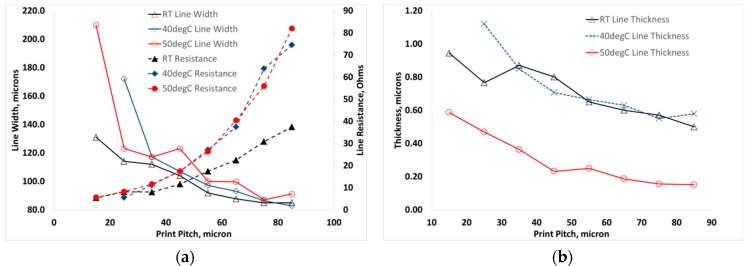
Effect of substrate temperature on (**a**) line width and resistance and (**b**) line thickness with varying print pitch.

On increasing the substrate temperature during printing, as shown in Figure 7a, little change in line width was observed however, printed features at 40 °C and 50 °C appeared to show an approximate doubling in resistance of the printed tracks. The thickness of the printed material at all temperatures showed the same trend of a non-linear increase in thickness with decreasing dot spacing over the investigated range. However, a significant decrease in the thickness was observed with a substrate temperature of 50 °C when compared with printing at room temperature and at 40 °C. The thickness showed around a 3-fold reduction at 50 °C when compared to the lower temperature results, as shown in Figure 7b.

**Figure 8:**
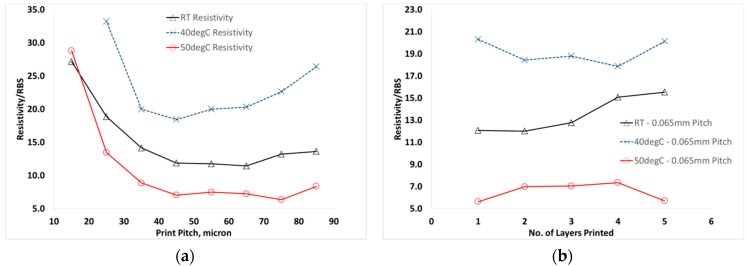
Effect of substrate temperature on (**a**) line resistivity with varying dot spacing and (**b**) line resistivity with number of layers printed.

As shown in Figure 8a, across all temperatures tested a similar trend of a largely constant resistivity value was observed up until a print pitch of 45 µm, after which an increasing resistivity with decreasing print pitch was observed. The printed material at 50 °C resulted in a reduced resistivity compared to lower temperatures, with resistivity of only 6–7.5 times RBS achieved between 75–45 µm dot spacing, due to the significant reduction in material thickness. With testing the effect of substrate temperature on overprinted tracks, a similar reduction in thickness and resistivity was observed at 50 °C, as shown in Figure 8b, where the resistivity approximately halved when compared to room temperature printed material. Considering previously reported work [21] on the effect of heating on the electrical properties, the effect of post-printing heat treatment for 15 min at varying temperatures was studied. Little effect on resistivity was found on heating to 50 °C, which gave only a slight improvement on room-temperature, whereas in the present work an approximate halving of resistivity was observed with 50 °C substrate temperature during printing. After 50 °C heat treatment of 10 overprinted layers a resistivity of 7 times RBS, which is comparable to the 5–7 times RBS typically observed for printed onto the 50 °C substrate observed in the present work. In order to achieve an approximate halving of resistivity, the results of the previous study [21] indicate that heating to between 120–140 °C for 15 min would be required. However, on further heating to 180 °C a further improvement in resistivity to around 3 times RBS was achieved but no observations of the effect of heating on material thickness were reported. 

In the present work, we propose that the reduction in film thickness observed at 50 °C is due to faster drying of the ink which enables dense packing of the AgNPs prior to extensive sintering due to chloride diffusion into the printed material. Diffusion of chloride and chemical sintering mainly occurs after drying of the film and then acts to coalesce and fused the dense packed NP film. At lower temperatures the slower drying of the ink enables diffusion of cholride and sintering to occur before drying to result in lower packing density and higher thickness of the film. In a similar study of AgNP sintering on a range of paper substrates [22], it was postulated that a denser and more ordered initial packing of the NPs resulting in lower resistivity of the films and disruption of the packing process resulted in lower density films, in [22] due to changes in substrate porosity, and higher resistivity. In the present work, the improvement in electrical properties on heating of the substrate suggests that further investigation of printing performance and sintering mechanism at substrate temperatures > 50 °C may bring further understanding and beneficial effects and should be investigated and compared to post-printing heat treatments in order to further optimise the electrical properties of the AgNP material.

## 3. Materials and Methods

### 3.1. Materials

Silver nitrate, 99.5% (AgNO_3_) was purchased from Alfa Aesar (Heysham, UK) and all the remaining chemicals or solvents including poly (acrylic acid) 1800 MW (PAA), triethanolamine (TEA) 99.0% (GC grade), ethanol (absolute, 99.8%), 1-propanol (anhydrous, 99.7%), ethylene glycol, 99.5% (GC grade) were purchased from Sigma Aldrich (Gillingham, UK) Glossy paper was purchased from EPSON (London, UK), milli-Q (Watford, UK) 18 MΩ deionised (DI) water was used in the synthesis process and ink preparation.

### 3.2. Synthesis of Ag Nanoparticles and Inkjet Formulation

In a typical synthesis procedure for PAA-capped silver Nanoparticles, 10.3 mL of TEA and 0.85 mmol of PAA were mixed in a flask containing 48 mL of DI water under stirring. Then, 10 g of silver nitrate dissolved in 10 mL of DI water was slowly added to the above mixture and the reaction mixture left under stirring at room temperature for 1 h. Once the silver nitrate was completely dispersed in the solution of PAA and TEA, the mixture turned pale cream to brown colour. Then, a temperature of 70 °C was applied to the mixture for 3 h under vigorous stirring. After 30 minutes, the solution turned a blackish brown colour. After 3 h vigorous stirring at 70 °C, the hotplate was removed, and the sample cooled to room temperature. The suspension was then kept under sonication for 1 hour using sonics and materials vibra cell by JENCONS scientific Ltd (Leighton Buzzard, UK) with an amplitude of 56%, full mode at room temperature. The obtained silver nanoparticles were washed 4 to 5 times with ethanol, then recovered by centrifugation and finally the pellet was dried at 70 °C to collect the solid silver nanoparticles (AgNPs). The obtained AgNPs were then used for the preparation of ink. The AgNP ink was prepared by addition of 30% by weight of the AgNPs to a mixture of water, ethylene glycol and alcohol followed by probe sonication for 30 minutes.

### 3.3. Characterisation of AgNPs and Inkjet Formulations

To determine the mean size and size distribution of nanoparticles, a JEOL (Welwyn Garden City, UK) 2010 200KV Transmission Electron Microscope (TEM) was used and the dispersed nanoparticle mean size and size distribution were measured using Dynamic Light Scattering (DLS) with a ZETASIZER Nano ZS from Malvern Instruments (Malvern, UK) Inkjet printing tests were performed using a Microfab (Plano, TX, USA) JETLAB4 printer with nozzles size of 80 µm and 50 µm. To determine the width, thickness and profile of the printed nanoparticle features, a Bruker (Coventry, UK) DektakXT Stylus Profiler was used, where the average of four or more thickness measurements were used. The resistivity of the printed tracks were determined by measuring the resistance of printed features using a Beha-Amprobe (Glottertal, Germany) Digital Multimeter and the well-known expression relating resistivity to Resistance, R=ρL/A=ρL/wt  (Ω), where ρ is the resistivity (Ω·m), *L* is the length of the line/track (m), *A* is the Area (m^2^), *w* is the width (m) and *t* is the thickness of the film/track (m) respectively. Optical microscopy images were obtained of the printed features using a Nikon (Minato, Tokyo, Japan) Eclipse LV150N. Surface Tension was measured at room temperature using Biolin Scientific (Gothenburg, Sweden) One Attension Theta Optical Tensiometer and Viscosity from TA Instruments (New Castle, DE, USA) AR2000 using the geometry 60 mm 1° cone with shear rate 0.1 to 1000/sec at 25 °C.

## 4. Conclusions

We have demonstrated stable inkjet printing of PAA-coated AgNP dispersions in water/alcohol/ethylene glycol formulations with sub-100 µm feature size. The lowest electrical resistivity from room-temperature printing was approximately 11 times resistivity of bulk silver. On increasing the thickness of printed material, by changing print pitch or by overprinting successive layers, an increase in resistivity beyond approximately 0.8 µm thickness was found due to less effective sintering. On heating the substrate temperature during printing to 50 °C an approximate halving of the resistivity was observed with the lowest electrical resistivity of 8.9 × 10^−8^ Ω·m, approaching five times resistivity of bulk silver. On heating to 50 °C a significant reduction in the material thickness was also observed and the thickness was around a third of that observed from printing at room-temperature
